# Supplementary material for: Exploiting convergent phenotypes to derive a pan-cancer cisplatin response gene expression signature
Source: NPJ Precis Oncol. 2023 Apr 19;7:38. doi: 10.1038/s41698-023-00375-y (PMC10115855; doi:10.1038/s41698-023-00375-y)
Supplement: Supplementary file 1 — Supplementary material without markup [file 41698_2023_375_MOESM1_ESM.pdf]

Supplementary Tables

Supplementary Table 1. Tissue of origin for all 429 epithelial-origin GDSC cell lines.

Tissue of origin	No. cell lines.
NSCLC adenocarcinoma	53
Breast	50
Large intestine	42
Ovary	33
Esophagus	30
Pancreas	30
Head and neck	28
Stomach	25
Bladder	18
Kidney	17
Mesothelium	16
Liver	15
NSCLC squamous	15
Cervix	14
Thyroid	14
Endometrium	11
Prostate	7
Biliary tract	5
Testis	3
Uterus	2
Adrenal gland	1

Supplementary Table 2. **Tissue of origin for DE comparison groups for fold 1.**

Drug Response	Tissue of origin	No. cell lines
Resistant	NSCLC adenocarcinoma	13
	Pancreas	9
	Breast	8
	Large intestine	7
	Mesothelium	5
	Kidney	4
	Ovary	4
	Stomach	3
	Thyroid	3
	Biliary Tract	2
	Endometrium	2
	Liver	2
	NSCLC squamous	2
	Esophagus	2
	Cervix	1
	Head and neck	1
	Prostate	1
Sensitive	Head and neck	10
	NSCLC adenocarcinoma	10
	Ovary	6
	Breast	5
	Large intestine	5
	Esophagus	5
	Bladder	4
	Kidney	4
	Pancreas	4
	Stomach	4
	Cervix	3
	Thyroid	3
	NSCLC squamous	2
	Prostate	1
	Testis	1
	Uterus	1

Supplementary Table 3. **Tissue of origin for DE comparison groups for fold 2.**

Drug Response	Tissue of origin	No. cell lines
Resistant	NSCLC adenocarcinoma	13
	Breast	9
	Pancreas	9
	Large intestine	5
	Mesothelium	5
	Stomach	5
	Esophagus	4
	Ovary	4
	Biliary Tract	2
	Head and neck	2
	Kidney	2
	Liver	2
	NSCLC squamous	2
	Thyroid	2
	Bladder	1
	Endometrium	1
	Prostate	1
Sensitive	Head and neck	11
	NSCLC adenocarcinoma	8
	Bladder	6
	Breast	5
	Large intestine	5
	Esophagus	5
	Ovary	5
	Stomach	5
	Cervix	3
	Kidney	3
	Pancreas	3
	NSCLC squamous	2
	Testis	2
	Thyroid	2
	Endometrium	1
	Prostate	1
	Uterus	1

Supplementary Table 4. **Tissue of origin for DE comparison groups for fold 3.**

Drug Response	Tissue of origin	No. cell lines
Resistant	NSCLC adenocarcinoma	13
	Pancreas	10
	Breast	8
	Large intestine	5
	Mesothelium	5
	Kidney	4
	Ovary	4
	Stomach	4
	Esophagus	3
	Thyroid	3
	Endometrium	2
	Head and neck	2
	Biliary Tract	1
	Bladder	1
	Cervix	1
	Liver	1
	NSCLC squamous	1
	Prostate	1
Sensitive	Ovary	10
	Head and neck	9
	Bladder	8
	NSCLC adenocarcinoma	6
	Esophagus	5
	Breast	4
	Cervix	4
	Kidney	4
	Pancreas	4
	Stomach	4
	Large intestine	3
	Thyroid	3
	Testis	2
	Endometrium	1
	NSCLC squamous	1
	Uterus	1

Supplementary Table 5. **Tissue of origin for DE comparison groups for fold 4.**

Drug Response	Tissue of origin	No. cell lines
Resistant	NSCLC adenocarcinoma	12
	Breast	10
	Large intestine	8
	Pancreas	6
	Mesothelium	5
	Ovary	5
	Thyroid	4
	Kidney	3
	Liver	3
	Esophagus	3
	Biliary Tract	2
	Head and neck	2
	Stomach	2
	Bladder	1
	Cervix	1
	Endometrium	1
	NSCLC squamous	1
Sensitive	Head and neck	9
	NSCLC adenocarcinoma	8
	Ovary	8
	Bladder	6
	Kidney	5
	Large intestine	5
	Stomach	5
	Cervix	4
	Esophagus	4
	Pancreas	3
	Thyroid	3
	Breast	2
	Testis	2
	Endometrium	1
	NSCLC squamous	1
	Prostate	1
	Uterus	1

Supplementary Table 6. **Tissue of origin for DE comparison groups for fold 5.**

Drug Response	Tissue of origin	No. cell lines
Resistant	NSCLC adenocarcinoma	13
	Pancreas	10
	Breast	8
	Large intestine	8
	Mesothelium	4
	Esophagus	4
	Stomach	4
	Kidney	3
	Ovary	3
	Thyroid	3
	Endometrium	2
	Biliary Tract	1
	Bladder	1
	Cervix	1
	Head and neck	1
	Liver	1
	NSCLC squamous	1
	Prostate	1
Sensitive	Head and neck	12
	NSCLC adenocarcinoma	8
	Ovary	8
	Stomach	6
	Bladder	5
	Breast	5
	Large intestine	5
	Esophagus	5
	Kidney	4
	Cervix	3
	Pancreas	2
	Endometrium	1
	NSCLC squamous	1
	Prostate	1
	Testis	1
	Thyroid	1

Supplementary Table 7. DE genes by fold. The SAM method consistently extracts more genes than limma or multtest. The intersection, however, is much smaller than either limma or multtest, showing significant filtering during the intersection step.

Fold	Method	No. Up-regulated Genes	No. Down-regulated Genes
1	SAM	1979	1083
	limma	181	322
	multtest	219	150
	intersection	59	58
2	SAM	1397	853
	limma	159	302
	multtest	139	115
	intersection	32	41
3	SAM	2290	1143
	limma	176	355
	multtest	247	173
	intersection	58	73
4	SAM	1904	1069
	limma	188	263
	multtest	237	147
	intersection	61	42
5	SAM	566	636
	limma	156	221
	multtest	93	87
	intersection	34	28

Supplementary Table 8. **NCCN Guideline versions used for assessing disease-site specific treatment guidelines.**

Disease Site	NCCN Guideline Version	Cisplatin Use	Notes for select circumstances
ACC	Neuroendocrine and Adrenal Tumors Version 3.2021	Yes	
BLCA	Bladder Cancer Version 3.2021.	Yes	
BRCA	Breast Cancer Version 5.2021	Select circumstances	Only for recurrent, unresectable triple negative BRCA with germline BRCA1/2 mutation
CESC	Cervical Cancer Version 1.2021	Yes	
CHOL	Hepatobiliary Cancers Version 5.2021	Yes	
COAD	Colon Cancer Version 2.2021	No	
ESCA	Esophageal and Esophagogastric Junction Cancers Version 3.2021	Yes	
HNSC	Head and Neck Cancers Version 3.2021	Yes	
KICH	Kidney Cancer Version 2.2022	No	
KIRP	Kidney Cancer Version 2.2022	No	
KIRC	Kidney Cancer Version 2.2022	No	
Kidney	Kidney Cancer Version 2.2022	No	
Renal Pelvis	Kidney Cancer Version 2.2022	No	
LIHC	Hepatobiliary Cancers Version 3.2021	No	
LUAD	Non-Small Cell Lung Cancer Version 5.2021	Yes	
LUSC	Non-Small Cell Lung Cancer Version 5.2021	Yes	
MESO	Malignant Pleural Mesothelioma Version 2.2021	Yes	
OV	Ovarian Cancer/Fallopian Tube Cancer/ Primary Peritoneal Cancer Version 1.2021	Yes	
PAAD	Pancreatic Adenocarcinoma Version 2.2021	Select circumstances	Only for BRCA1/2 or PALB2 mutations
PRAD	Prostate Cancer Version 2.2021	No	
READ	Rectal Cancer Version 1.2021	No	
STAD	Gastric cancer Version 3.2021	Yes	
THCA	Thyroid Carcinoma Version 1.2021	Select circumstances	Only as adjuvant/radiosensitizer for anaplastic carcinoma
THYM	Thymomas and Thymic Carcinomas Version 1.2021	Yes	
UCEC	Uterine Neoplasms Version 3.2021	Yes	

Supplementary Table 9. Resulting coefficients for multivariate model trained in Figure 6.

Gene	Coefficient Estimate	Exponentiated Coefficient Estimate	Coefficient Standard Error
C15orf41	-2.2185	0.1088	1.3481
FKBP14	-1.5948	0.2029	1.0083
PSAT1	-1.8362	0.1594	1.5889
C1QBP	0.2797	1.3227	1.0234

Supplementary Table 10. Resulting coefficients for multivariate model trained in Supplementary Figure 17.

Gene	Coefficient Estimate	Exponentiated Coefficient Estimate	Coefficient Standard Error
C15orf41	-0.6801	0.5066	0.3784
FKBP14	-0.3735	0.6883	0.5040
PSAT1	-0.2059	0.8139	0.5684

Supplementary Figures

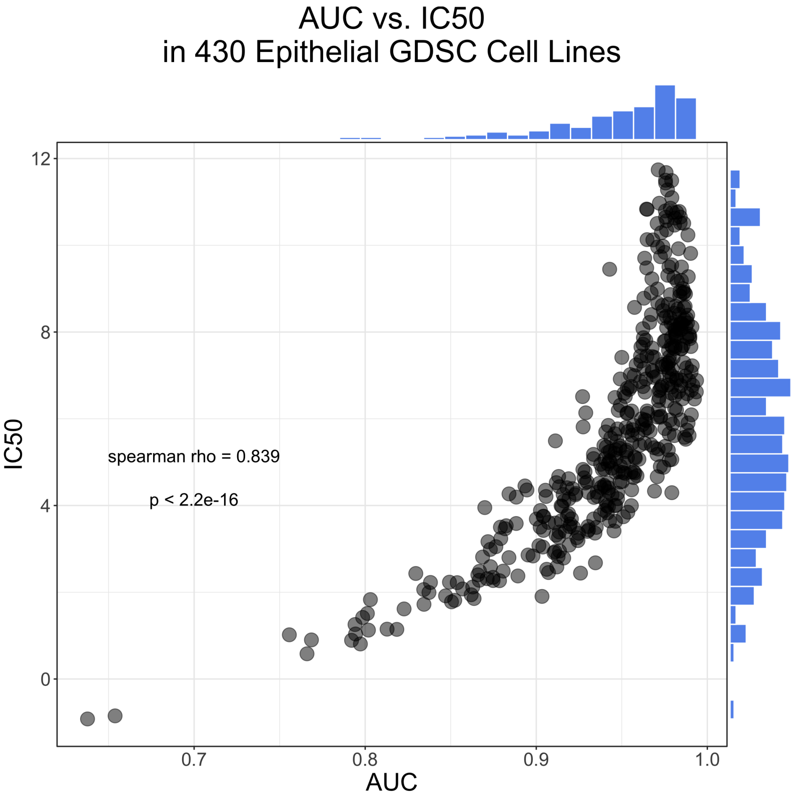

Supplementary Figure 1. Correlation between AUC and IC50 drug response metrics for epithelial-based cancer cell lines in the Genomics of Drug Discovery in Cancer (GDSC) dataset.

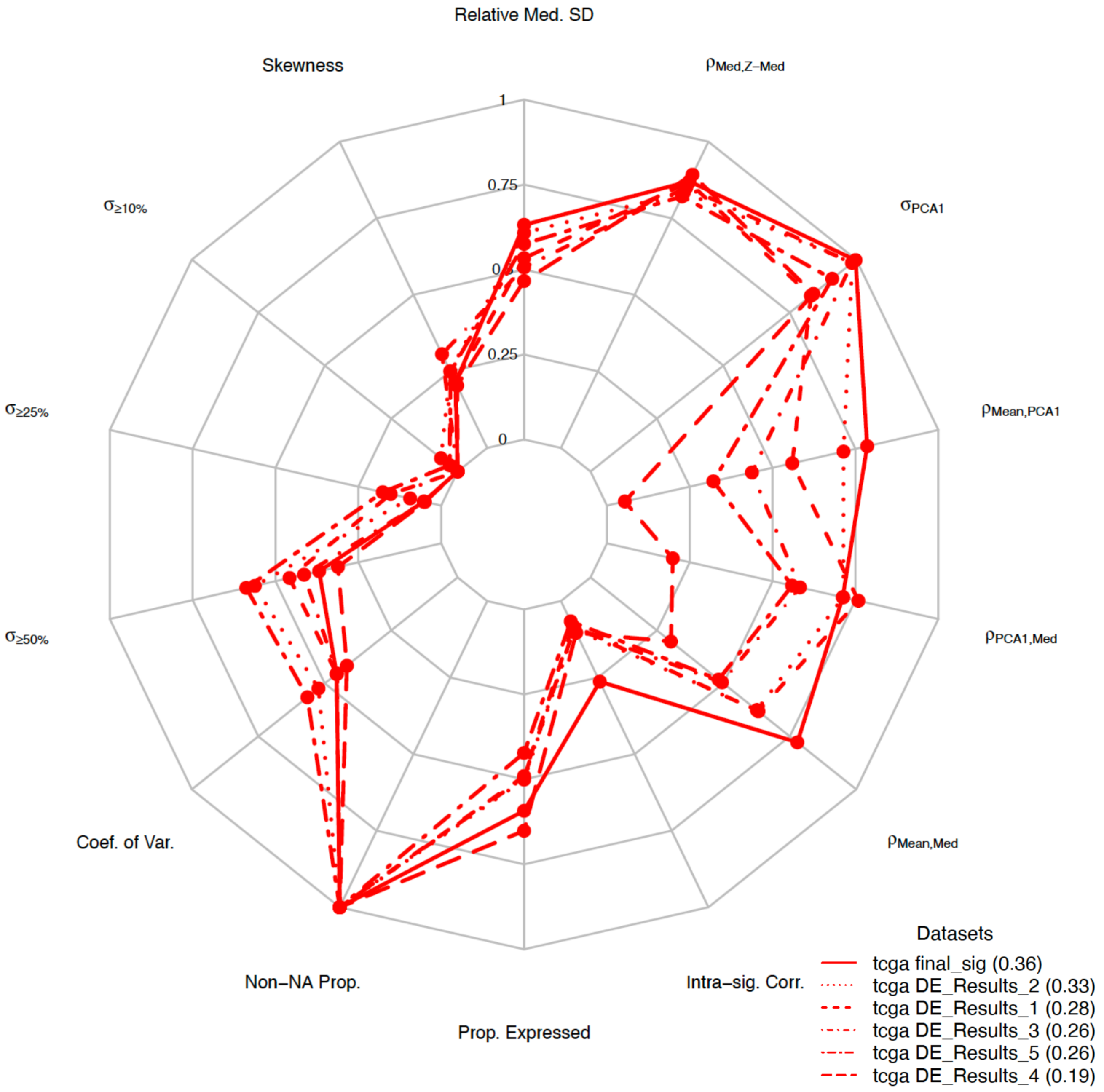

Supplementary Figure 2. Quality control metrics comparing differential expression results to the final gene signature using sigQC[26,27]. CisSig is compared to the folds of differential gene expression analysis, displaying results using a radar plot. Created with BioRender.com.

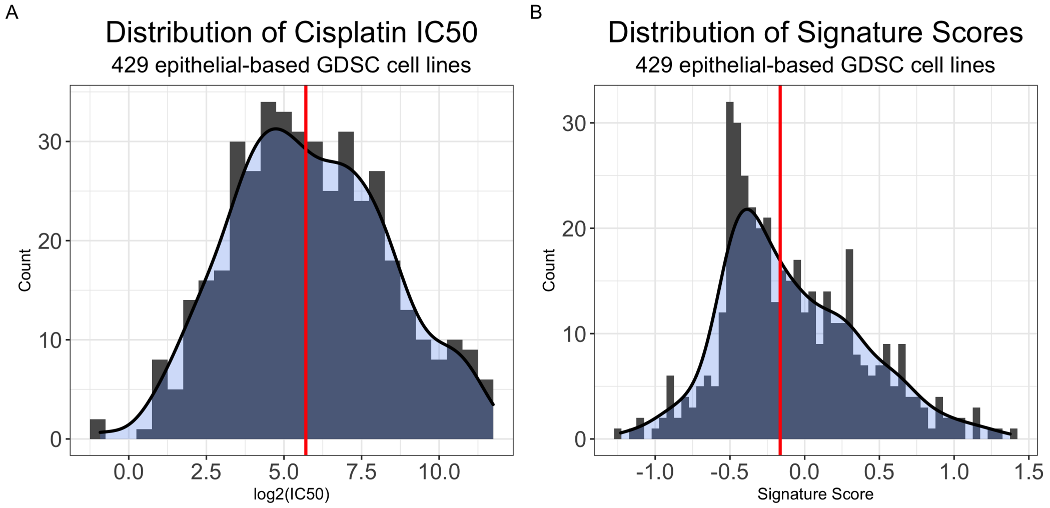

Supplementary Figure 3. Cisplatin IC50 (log2-transformed) in epithelial-origin GDSC cell lines is relatively normally distributed, while CisSig Score has a slight right skew. A. Distribution of CisSig across 429 epithelial-based GDSC cell lines, using a histogram (gray) and kernel density estimation (blue). Median score marked by red vertical line. CisSig score is calculated as a cell line's median normalized expression of CisSig genes listed in A. **B.** Distribution of cisplatin IC50 across 429 epithelial-based GDSC cell lines, using a histogram (gray) and kernel density estimation (blue). Median IC50 marked by red vertical line.

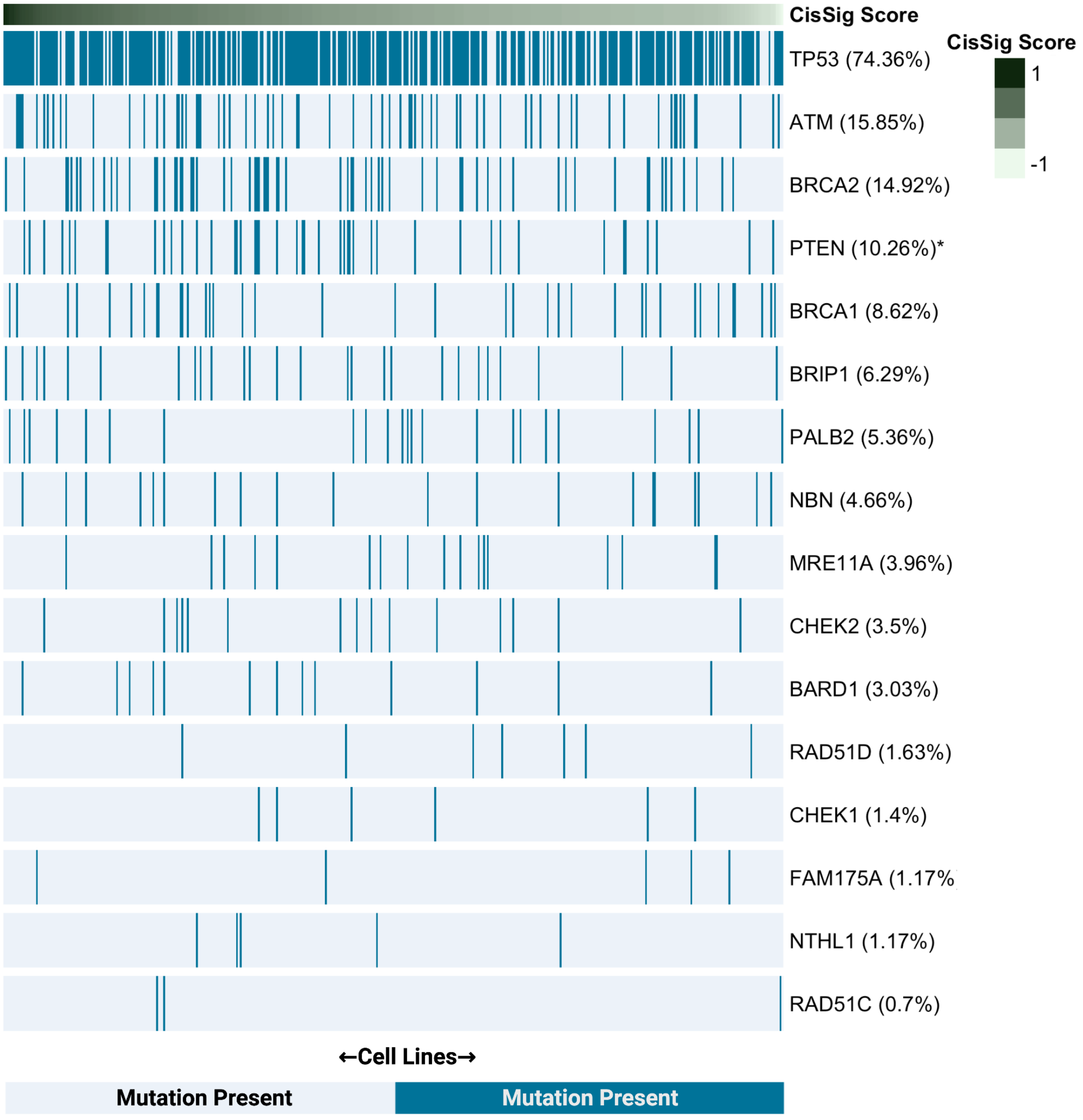

Supplementary Figure 4. CisSig Score is not associated with mutation status in most genes related to DNA damage response. A heatmap showing the mutation status for a variety of genes related to DNA damage response, in all epithelial based cell lines in the GDSC dataset. A cell line with the presence of a missense, frameshift, exonic splicing silencer, nonsense, inframe, or stop lost mutation is denoted as having a "mutation present" in that gene. Columns are ordered by CisSig Score, and rows are ordered by the frequency of a gene's mutation within these cell lines. Mutation frequency is denoted in parentheses to the right of each gene name. After Bonferroni correction for multiple hypothesis testing, there is only a statistical association (corrected p-value < 0.05 using chi-square test) for the PTEN gene (marked with an asterisk), where cell lines within the top half of CisSig scores are more likely to have a PTEN mutation than those within the bottom half of CisSig scores. Created with BioRender.com.

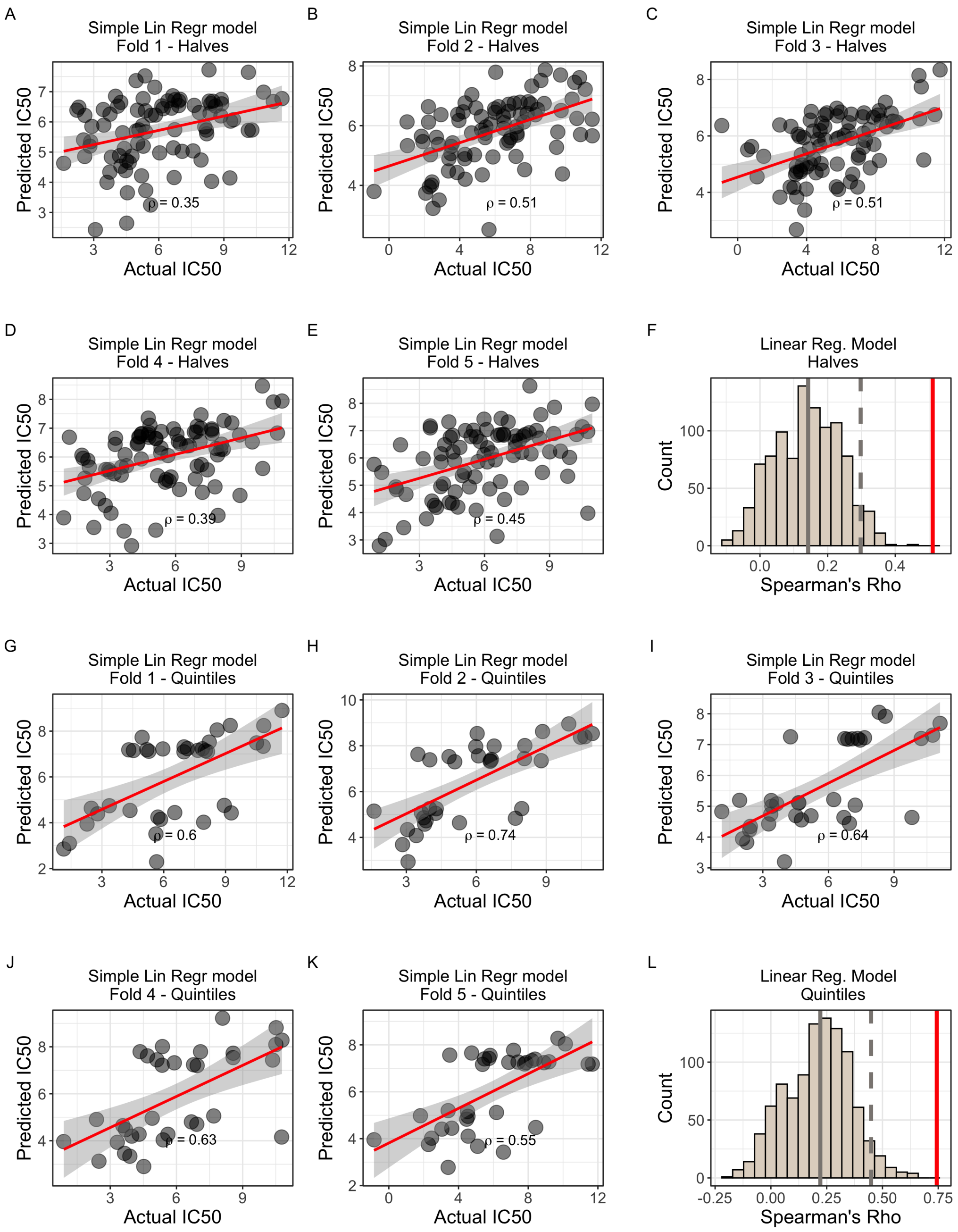

Supplementary Figure 5. Modeling IC50 response using CisSig Score to predict IC50 in GDSC with simple linear regression. A-E. Predicted vs. Actual IC50 for validation sets of folds 1-5 for models built with all 429 cell lines. **F.** Null distribution of modeling metrics using 1000 random gene signatures with the same length as CisSig and the model described in **A-E**. CisSig's performance (red solid line) is within the top 5% of the null distribution (cutoff at gray dashed line). Gray solid line represents median of null distribution. **G-K.** Predicted vs. Actual IC50 for validation sets of folds 1-5 for models built using cell lines in the top and bottom 20% of cisplatin IC50. **F.** Null distribution of modeling metrics using 1000 random gene signatures with the same length as CisSig and the model described in **G-K**. CisSig's performance (red solid line) is compared to the 95% confidence interval (gray dashed line) of the null distribution. Gray solid line represents median of null distribution.

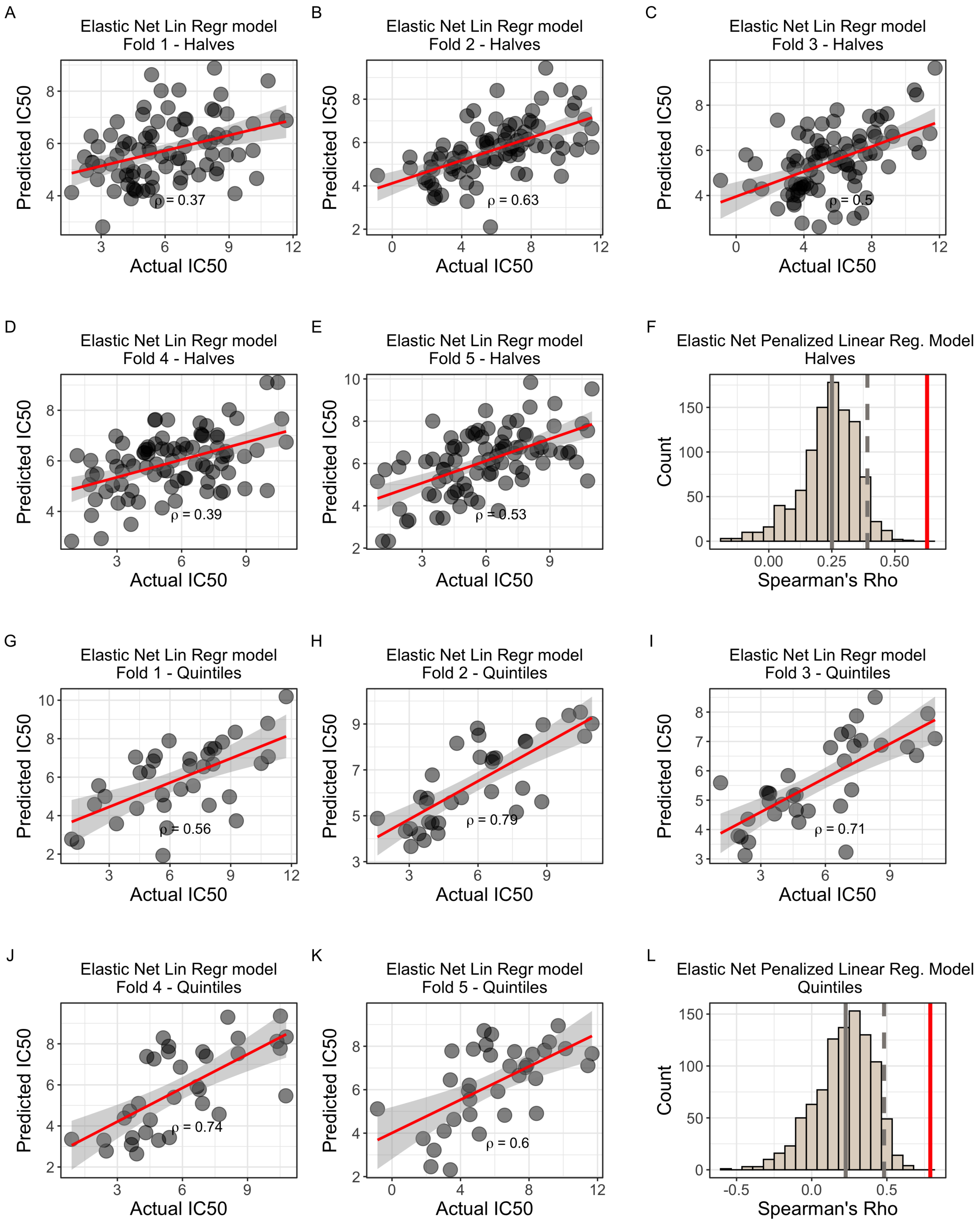

Supplementary Figure 6. Modeling IC50 response using individual CisSig genes to predict IC50 in GDSC with elastic net penalized linear regression. A-E. Predicted vs. Actual IC50 for validation sets of folds 1-5 for models built with all 429 cell lines. **F.** Null distribution of modeling metrics using 1000 random gene signatures with the same length as CisSig and the model described in **A-E**. CisSig's performance (red solid line) is within the top 5% of the null distribution (cutoff at gray dashed line). Gray solid line represents median of null distribution. **G-K.** Predicted vs. Actual IC50 for validation sets of folds 1-5 for models built using cell lines in the top and bottom 20% of cisplatin IC50. **F.** Null distribution of modeling metrics using 1000 random gene signatures with the same length as CisSig and the model described in **G-K**. CisSig's performance (red solid line) is compared to the 95% confidence interval (gray dashed line) of the null distribution. Gray solid line represents median of null distribution.

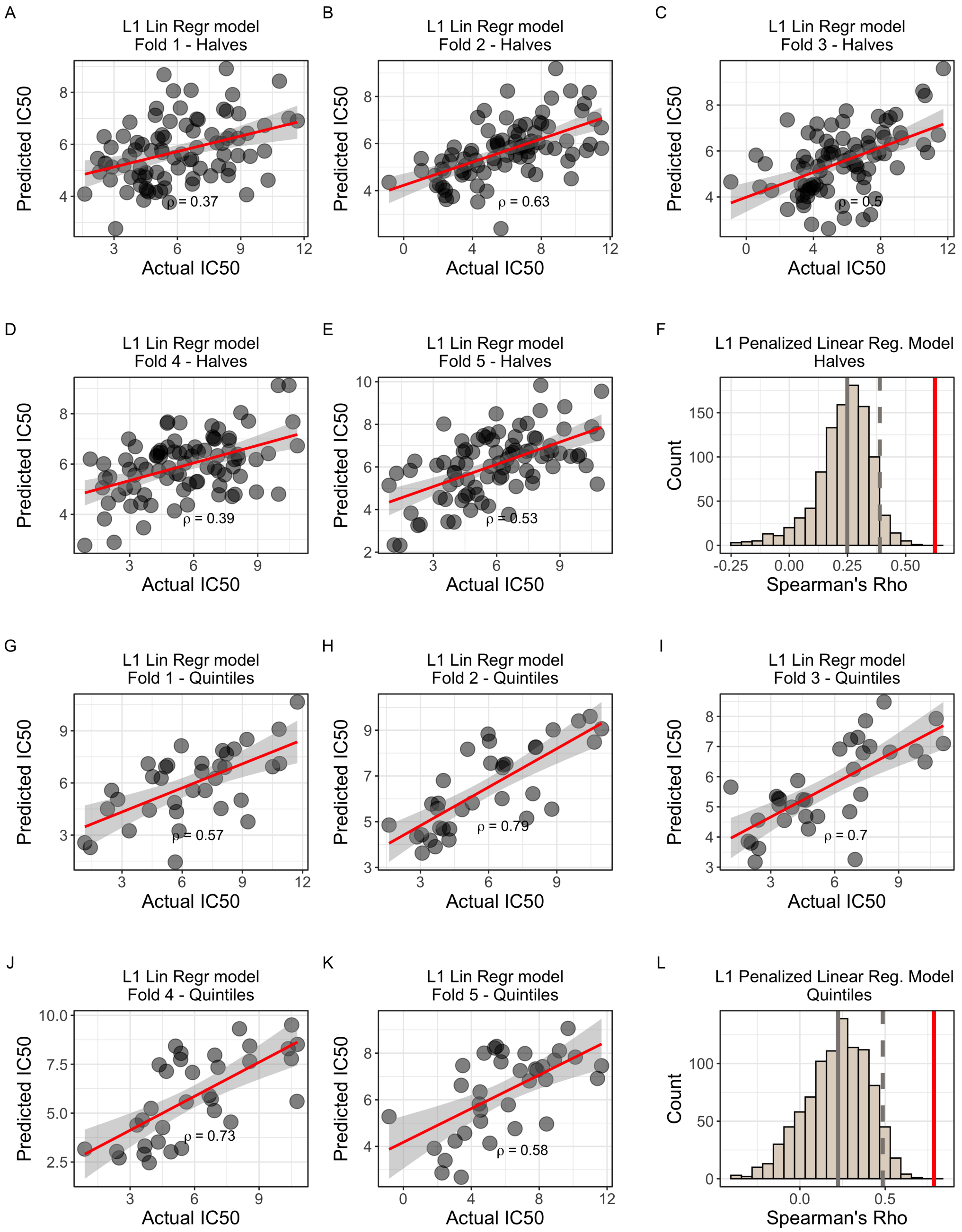

Supplementary Figure 7. Modeling IC50 response using individual CisSig genes to predict IC50 in GDSC with L1 penalized linear regression. A-E. Predicted vs. Actual IC50 for validation sets of folds 1-5 for models built with all 429 cell lines. **F.** Null distribution of modeling metrics using 1000 random gene signatures with the same length as CisSig and the model described in **A-E**. CisSig's performance (red solid line) is within the top 5% of the null distribution (cutoff at gray dashed line). Gray solid line represents median of null distribution. **G-K.** Predicted vs. Actual IC50 for validation sets of folds 1-5 for models built using cell lines in the top and bottom 20% of cisplatin IC50. **F.** Null distribution of modeling metrics using 1000 random gene signatures with the same length as CisSig and the model described in **G-K**. CisSig's performance (red solid line) is compared to the 95% confidence interval (gray dashed line) of the null distribution. Gray solid line represents median of null distribution.

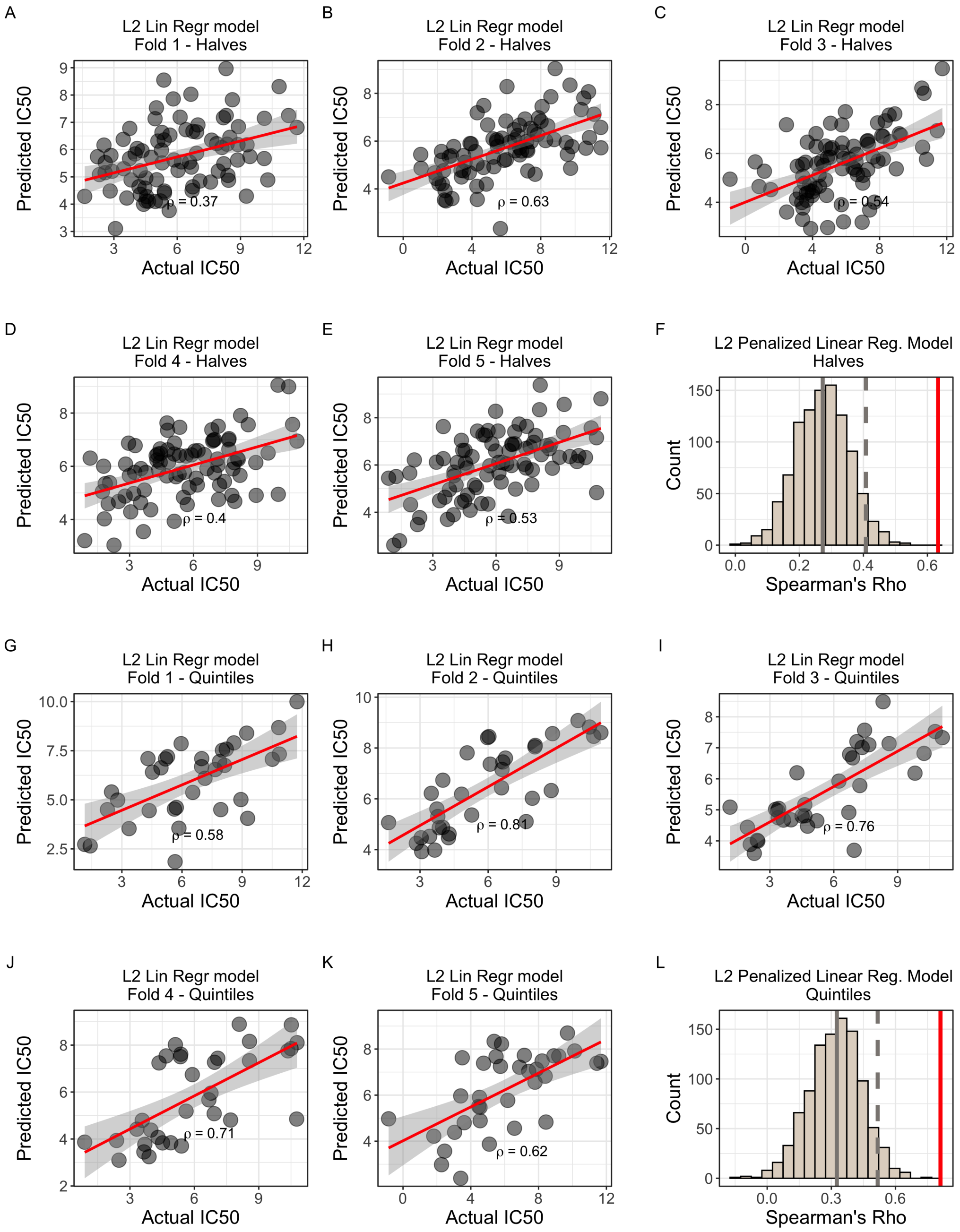

Supplementary Figure 8. Modeling IC50 response using individual CisSig genes to predict IC50 in GDSC with L2 penalized linear regression. A-E. Predicted vs. Actual IC50 for validation sets of folds 1-5 for models built with all 429 cell lines. **F.** Null distribution of modeling metrics using 1000 random gene signatures with the same length as CisSig and the model described in **A-E**. CisSig's performance (red solid line) is within the top 5% of the null distribution (cutoff at gray dashed line). Gray solid line represents median of null distribution. **G-K.** Predicted vs. Actual IC50 for validation sets of folds 1-5 for models built using cell lines in the top and bottom 20% of cisplatin IC50. **F.** Null distribution of modeling metrics using 1000 random gene signatures with the same length as CisSig and the model described in **G-K**. CisSig's performance (red solid line) is compared to the 95% confidence interval (gray dashed line) of the null distribution. Gray solid line represents median of null distribution.

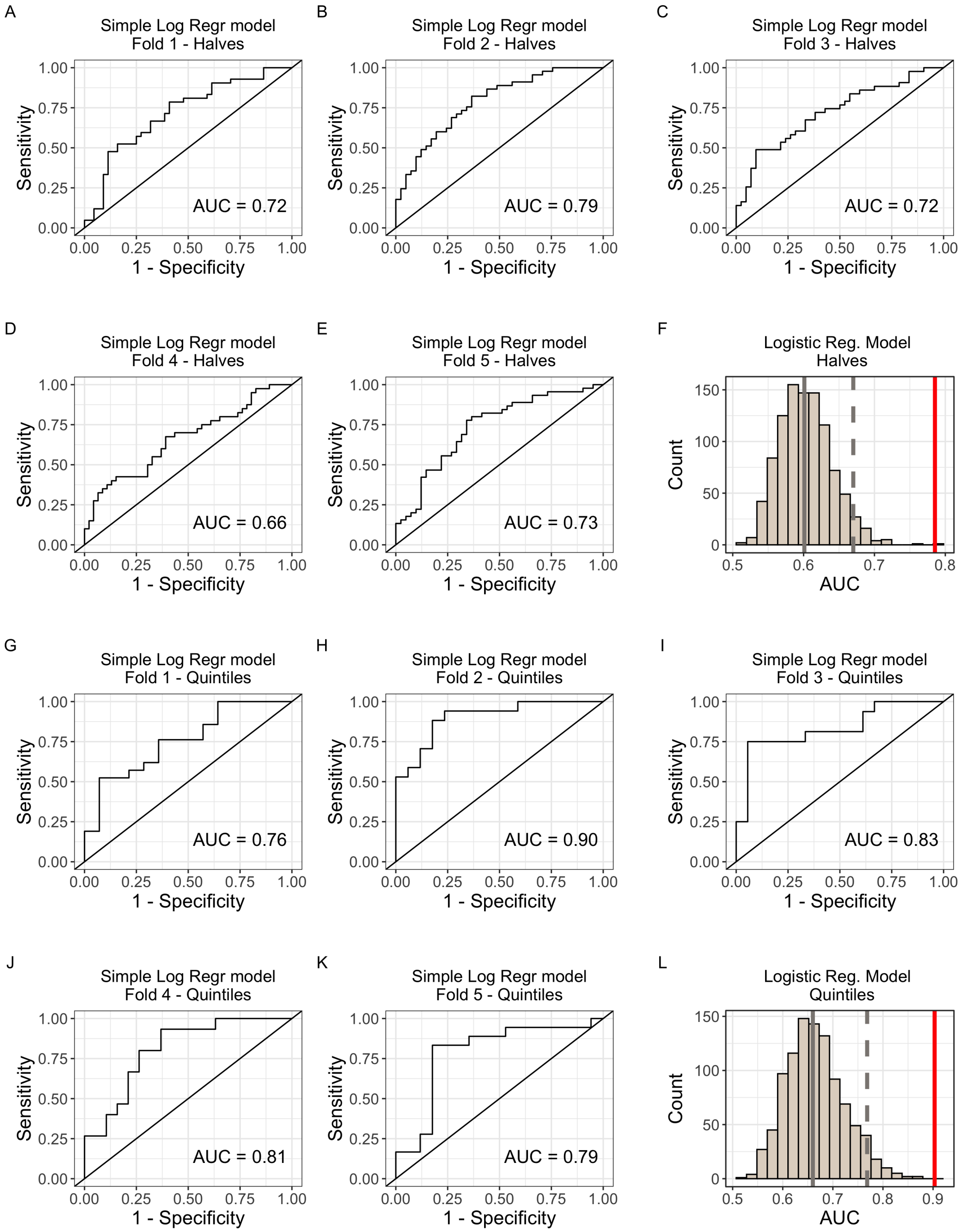

Supplementary Figure 9. Modeling IC50 response using CisSig score to predict IC50 class in GDSC with simple logistic regression. A-E. AUC for validation sets of folds 1-5 for models built with all 429 cell lines. **F.** Null distribution of modeling metrics using 1000 random gene signatures with the same length as CisSig and the model described in **A-E**. CisSig's performance (red solid line) is within the top 5% of the null distribution (cutoff at gray dashed line). Gray solid line represents median of null distribution. **G-K.** AUC for validation sets of folds 1-5 for models built using cell lines in the top and bottom 20% of cisplatin IC50. **F.** Null distribution of modeling metrics using 1000 random gene signatures with the same length as CisSig and the model described in **G-K**. CisSig's performance (red solid line) is compared to the 95% confidence interval (gray dashed line) of the null distribution. Gray solid line represents median of null distribution.

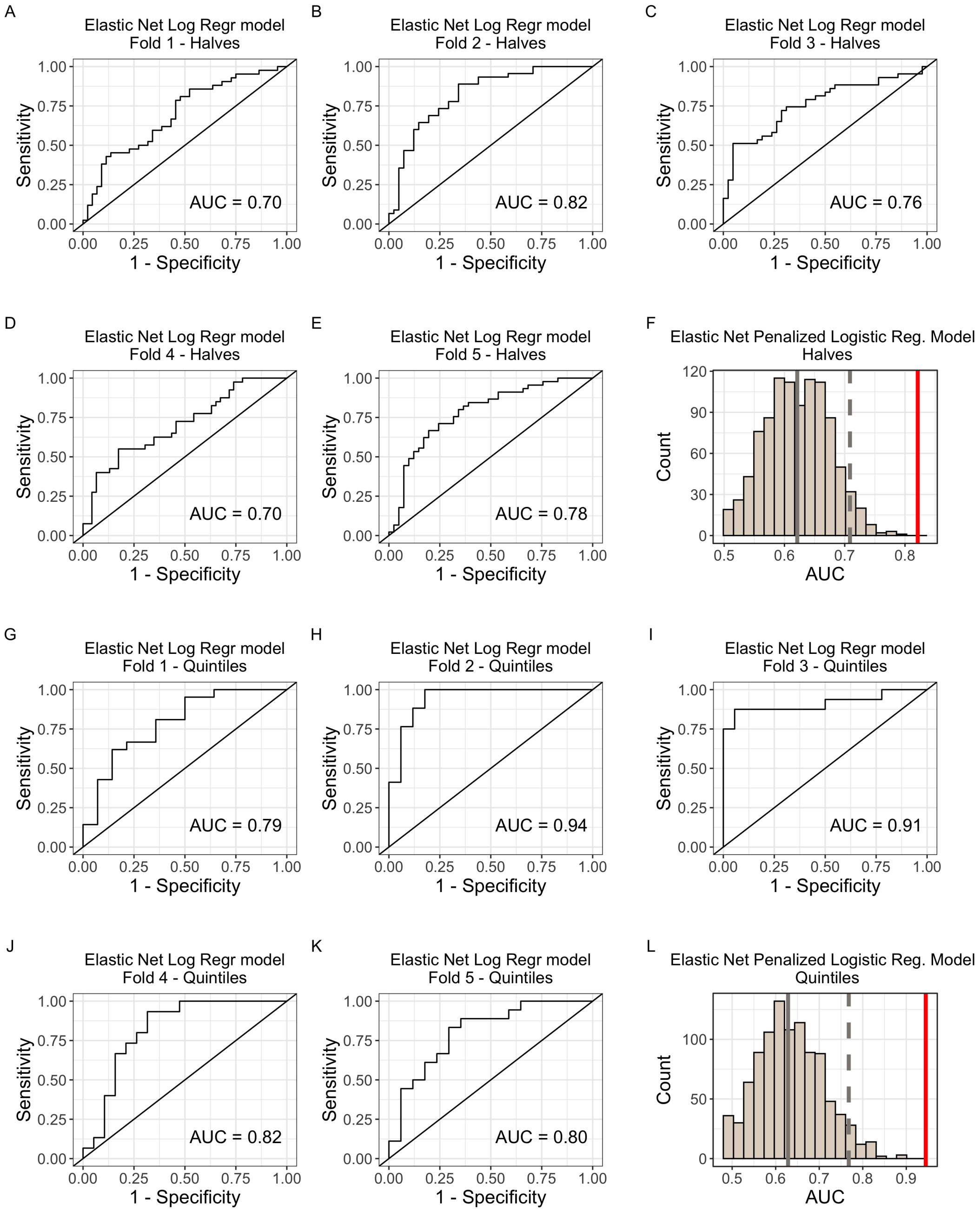

Supplementary Figure 10. Modeling IC50 response using individual CisSig genes to predict IC50 class in GDSC with elastic net penalized logistic regression. A-E. AUC for validation sets of folds 1-5 for models built with all 429 cell lines. **F.** Null distribution of modeling metrics using 1000 random gene signatures with the same length as CisSig and the model described in **A-E**. CisSig's performance (red solid line) is within the top 5% of the null distribution (cutoff at gray dashed line). Gray solid line represents median of null distribution. **G-K.** AUC for validation sets of folds 1-5 for models built using cell lines in the top and bottom 20% of cisplatin IC50. **F.** Null distribution of modeling metrics using 1000 random gene signatures with the same length as CisSig and the model described in **G-K**. CisSig's performance (red solid line) is compared to the 95% confidence interval (gray dashed line) of the null distribution. Gray solid line represents median of null distribution.

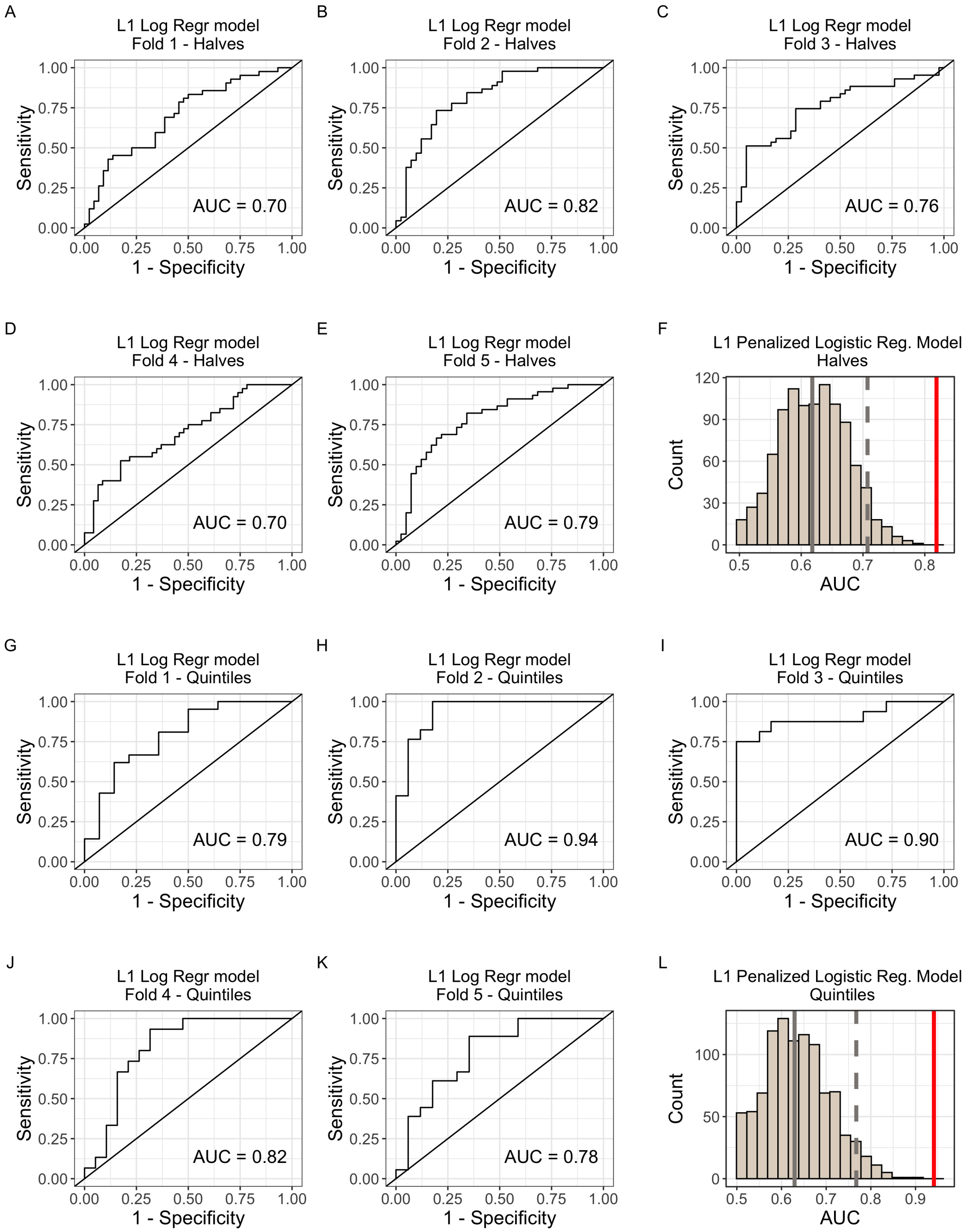

Supplementary Figure 11. Modeling IC50 response using individual CisSig genes to predict IC50 class in GDSC with L1 penalized logistic regression. A-E. AUC for validation sets of folds 1-5 for models built with all 429 cell lines. **F.** Null distribution of modeling metrics using 1000 random gene signatures with the same length as CisSig and the model described in **A-E**. CisSig's performance (red solid line) is within the top 5% of the null distribution (cutoff at gray dashed line). Gray solid line represents median of null distribution. **G-K.** AUC for validation sets of folds 1-5 for models built using cell lines in the top and bottom 20% of cisplatin IC50. **F.** Null distribution of modeling metrics using 1000 random gene signatures with the same length as CisSig and the model described in **G-K**. CisSig's performance (red solid line) is compared to the 95% confidence interval (gray dashed line) of the null distribution. Gray solid line represents median of null distribution.

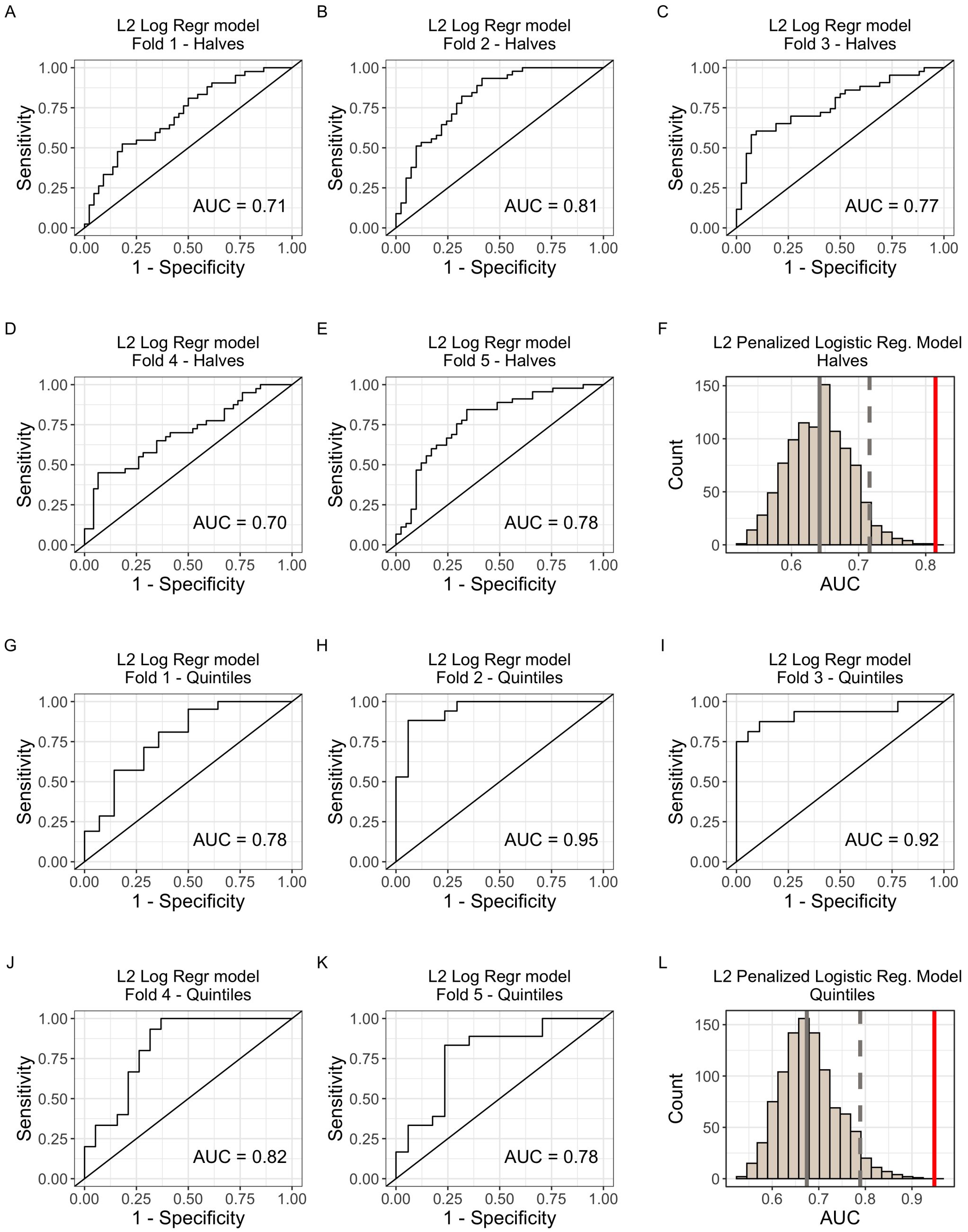

Supplementary Figure 12. Modeling IC50 response using individual CisSig genes to predict IC50 class in GDSC with L2 penalized logistic regression. A-E. AUC for validation sets of folds 1-5 for models built with all 429 cell lines. **F.** Null distribution of modeling metrics using 1000 random gene signatures with the same length as CisSig and the model described in **A-E**. CisSig's performance (red solid line) is within the top 5% of the null distribution (cutoff at gray dashed line). Gray solid line represents median of null distribution. **G-K.** AUC for validation sets of folds 1-5 for models built using cell lines in the top and bottom 20% of cisplatin IC50. **F.** Null distribution of modeling metrics using 1000 random gene signatures with the same length as CisSig and the model described in **G-K**. CisSig's performance (red solid line) is compared to the 95% confidence interval (gray dashed line) of the null distribution. Gray solid line represents median of null distribution.

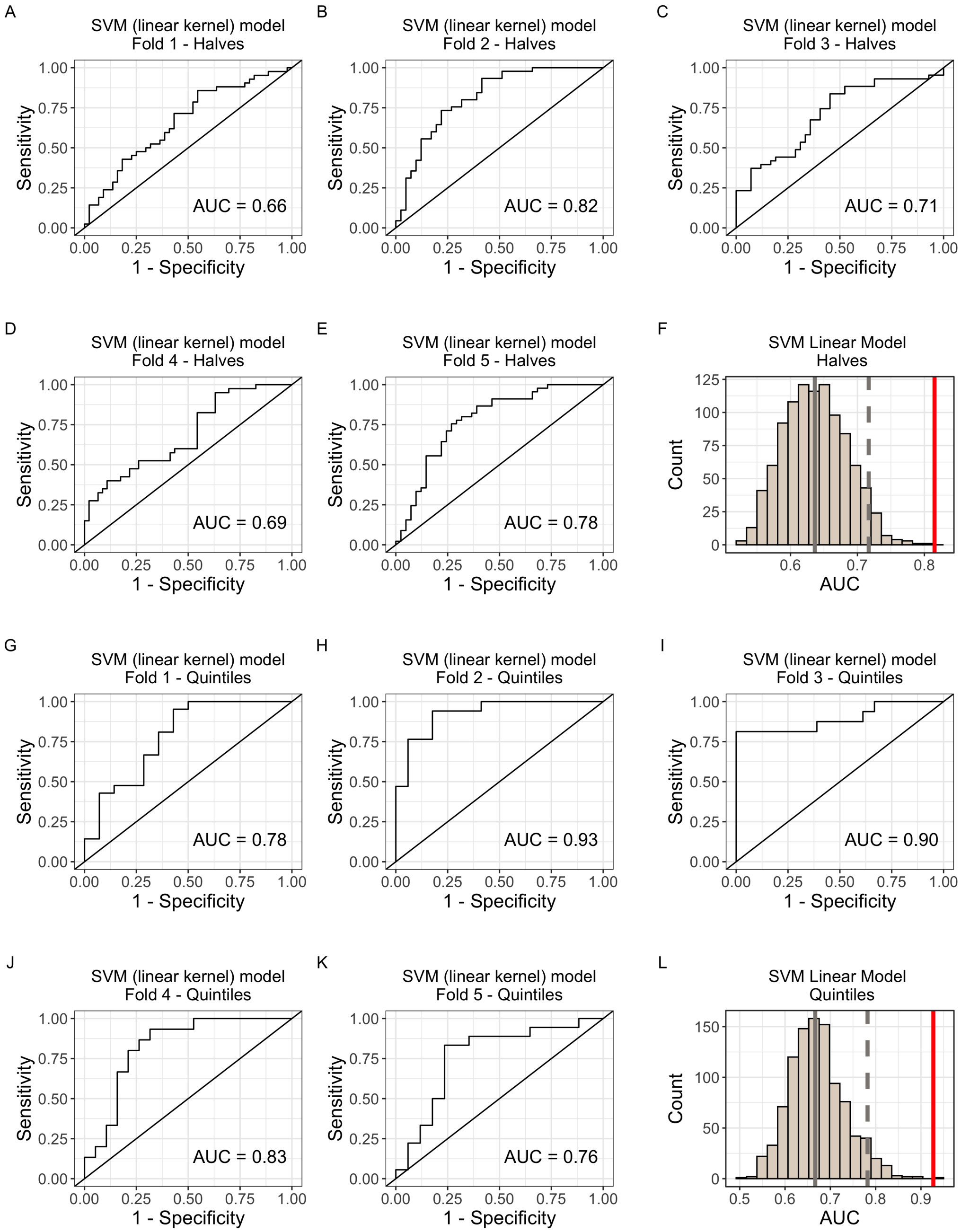

Supplementary Figure 13. Modeling IC50 response using individual CisSig genes to predict IC50 class in GDSC with support vector machine modeling (linear kernel). A-E. AUC for validation sets of folds 1-5 for models built with all 429 cell lines. **F.** Null distribution of modeling metrics using 1000 random gene signatures with the same length as CisSig and the model described in **A-E**. CisSig's performance (red solid line) is within the top 5% of the null distribution (cutoff at gray dashed line). Gray solid line represents median of null distribution. **G-K.** AUC for validation sets of folds 1-5 for models built using cell lines in the top and bottom 20% of cisplatin IC50. **F.** Null distribution of modeling metrics using 1000 random gene signatures with the same length as CisSig and the model described in **G-K**. CisSig's performance (red solid line) is compared to the 95% confidence interval (gray dashed line) of the null distribution. Gray solid line represents median of null distribution.

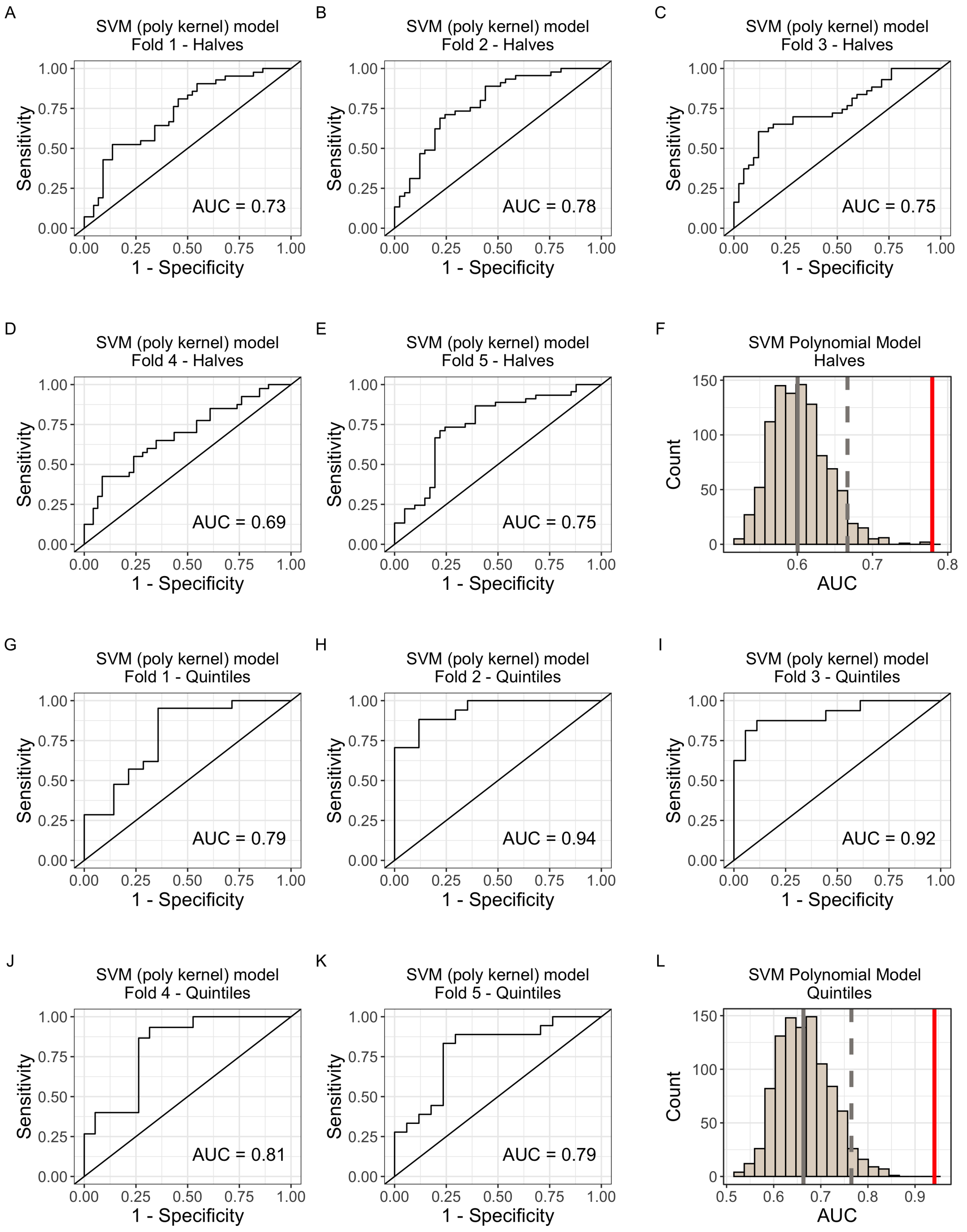

Supplementary Figure 14. Modeling IC50 response using individual CisSig genes to predict IC50 class in GDSC with support vector machine modeling (polynomial kernel). A-E. AUC for validation sets of folds 1-5 for models built with all 429 cell lines. **F.** Null distribution of modeling metrics using 1000 random gene signatures with the same length as CisSig and the model described in **A-E**. CisSig's performance (red solid line) is within the top 5% of the null distribution (cutoff at gray dashed line). Gray solid line represents median of null distribution. **G-K.** AUC for validation sets of folds 1-5 for models built using cell lines in the top and bottom 20% of cisplatin IC50. **F.** Null distribution of modeling metrics using 1000 random gene signatures with the same length as CisSig and the model described in **G-K**. CisSig's performance (red solid line) is compared to the 95% confidence interval (gray dashed line) of the null distribution. Gray solid line represents median of null distribution.

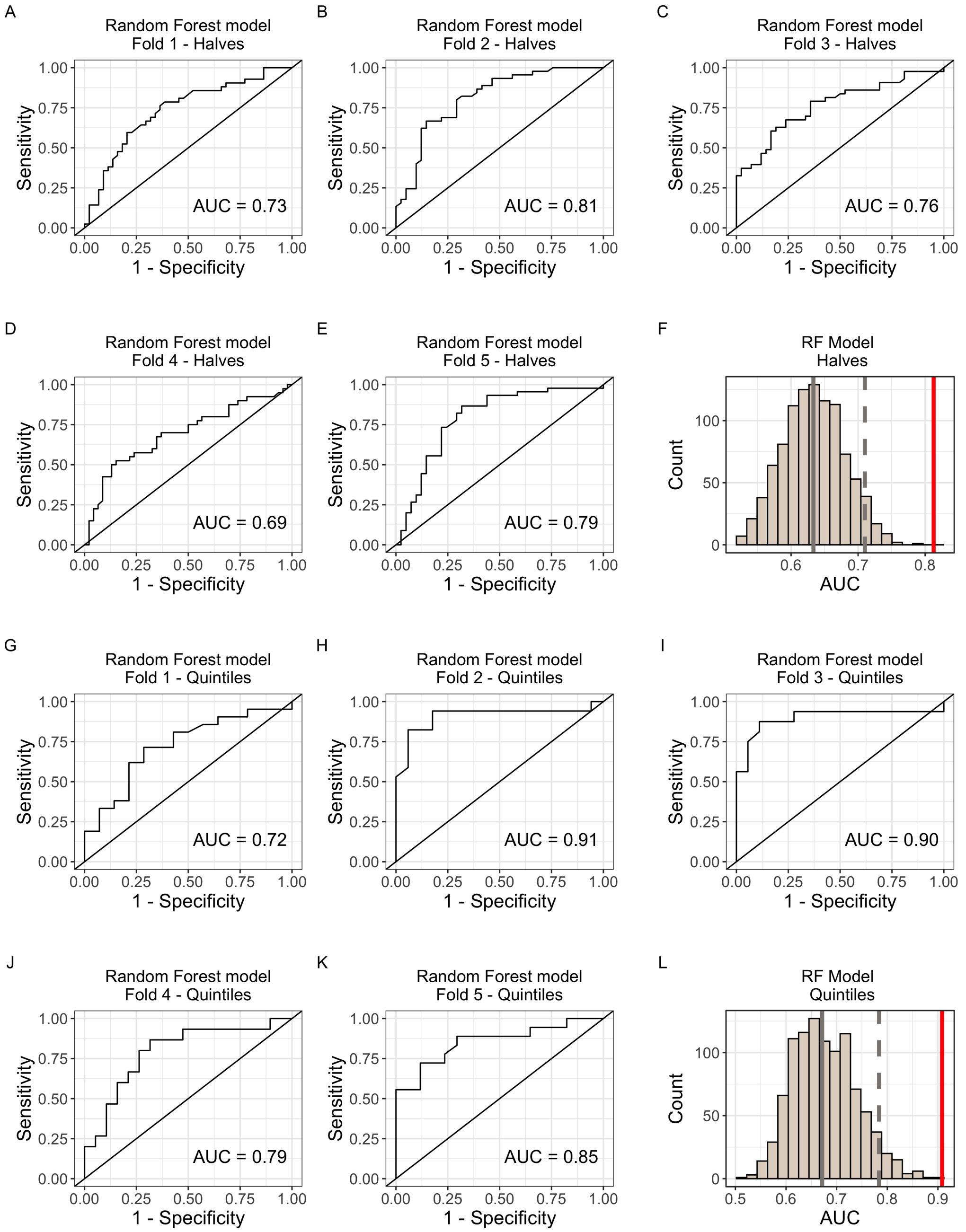

Supplementary Figure 15. Modeling IC50 response using individual CisSig genes to predict IC50 class in GDSC with random forest modeling. A-E. AUC for validation sets of folds 1-5 for models built with all 429 cell lines. **F.** Null distribution of modeling metrics using 1000 random gene signatures with the same length as CisSig and the model described in **A-E**. CisSig's performance (red solid line) is within the top 5% of the null distribution (cutoff at gray dashed line). Gray solid line represents median of null distribution. **G-K.** AUC for validation sets of folds 1-5 for models built using cell lines in the top and bottom 20% of cisplatin IC50. **F.** Null distribution of modeling metrics using 1000 random gene signatures with the same length as CisSig and the model described in **G-K**. CisSig's performance (red solid line) is compared to the 95% confidence interval (gray dashed line) of the null distribution. Gray solid line represents median of null distribution.

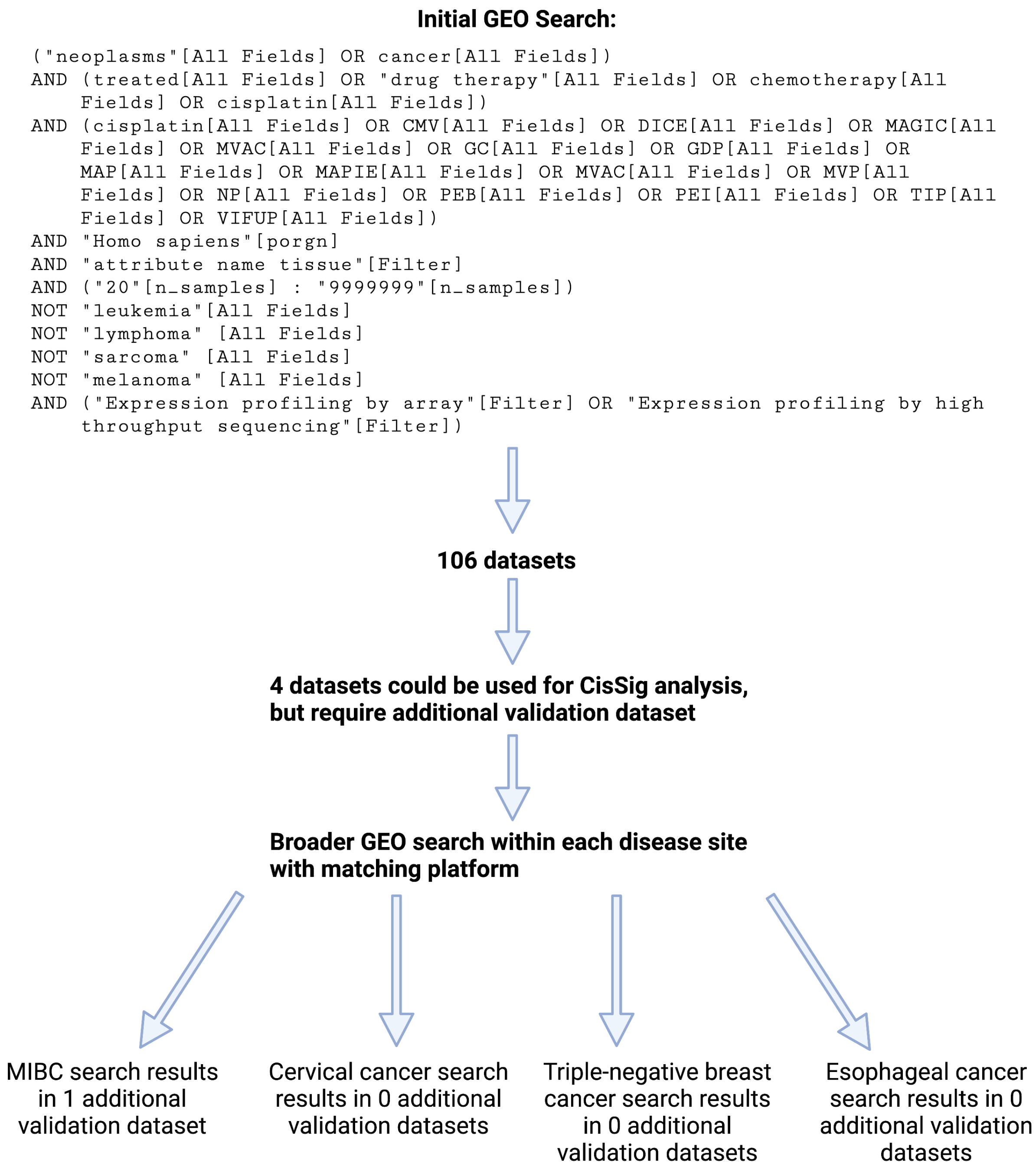

Supplementary Figure 16. Description of GEO search workflow. Last updated December 31, 2022. Created with BioRender.com.

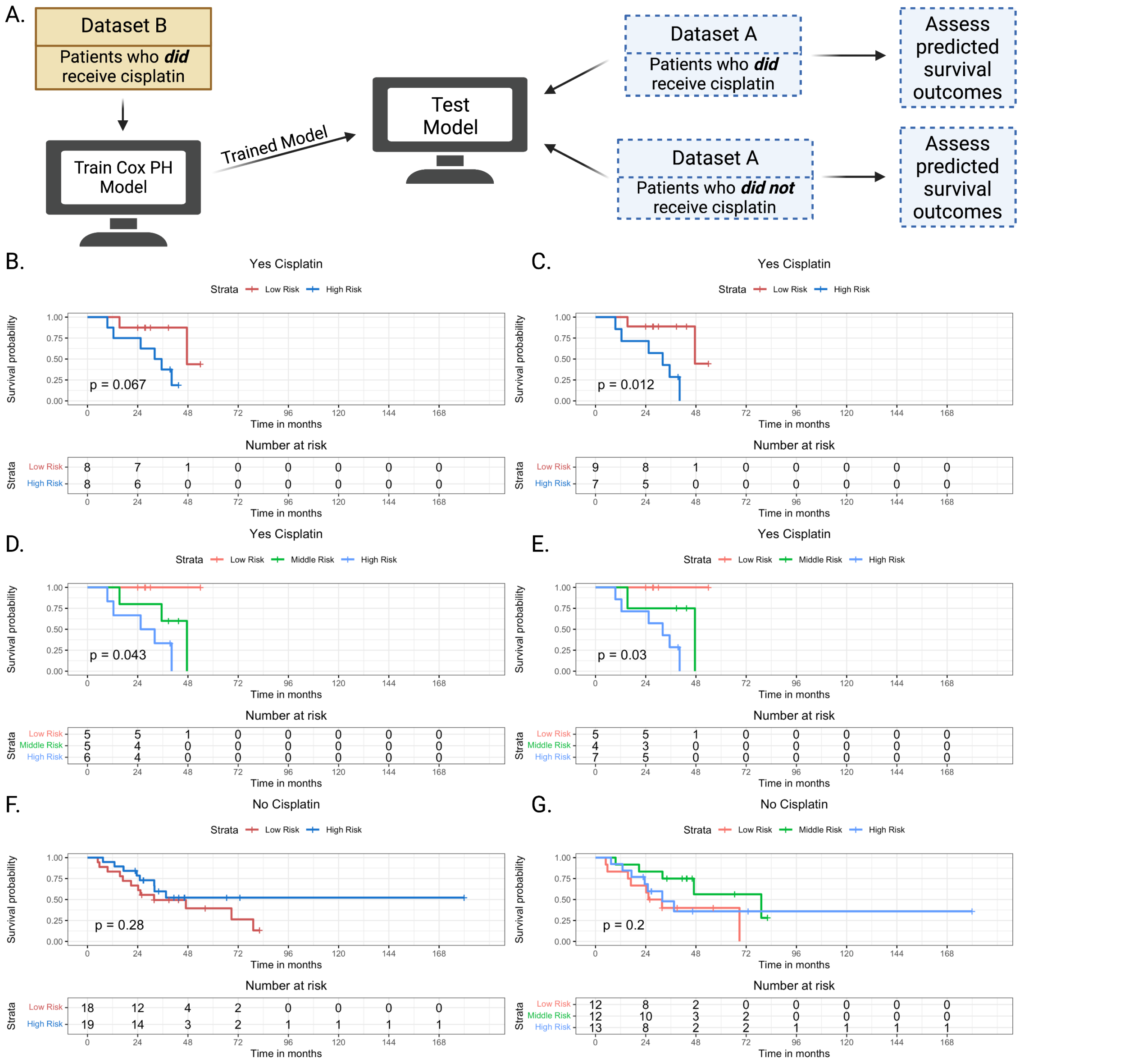

Supplementary Figure 17. CisSig-trained model is predictive in patients who have received cisplatin, but lacks signal in patients who have not received cisplatin. A. Schematic description of model training and testing, where model is trained using patients who did receive cisplatin-containing treatment from Dataset B. Testing of the trained model is done using patients from the Dataset A who did not receive cisplatin-containing treatment and patients from the Dataset A who did receive cisplatin-containing treatment. **B.** Test samples that did receive cisplatin-containing treatment are separated into groups of "high" and "low risk" based on the model's predictions using a median cutoff. Kaplan-meier curves show a separation between the two groups (that is just outside of range for statistical significance with a cutoff of p = 0.5). **C.** The same analysis shown in **B**, using an optimal cutpoint (determined by chi-square statistic) instead of median to separate the cohorts. **D-E.** The same analyses shown in **B-C**, separating the groups into "high", "middle", and "low risk" groups using tertiles and the optimal two cutpoints, respectively. **F-G.** The same analyses shown in **B** and **D**, using samples from Dataset A that did not receive cisplatin-containing treatment, demonstrating no significant separation between the two groups. Created with BioRender.com.
