# Supplementary material for: Exploiting convergent phenotypes to derive a pan-cancer cisplatin response gene expression signature
Source: NPJ Precis Oncol. 2023 Apr 19;7:38. doi: 10.1038/s41698-023-00375-y (PMC10115855; doi:10.1038/s41698-023-00375-y)
Supplement: Supplementary file 2 — REPORTING SUMMARY [file 41698_2023_375_MOESM2_ESM.pdf]

nature portfolio

Corresponding author(s): Jacob Scott
Andrew Dhawan

Last updated by author(s): Jan 10, 2023

Reporting Summary

Nature Portfolio wishes to improve the reproducibility of the work that we publish. This form provides structure for consistency and transparency in reporting. For further information on Nature Portfolio policies, see our Editorial Policies and the Editorial Policy Checklist.

Statistics

For all statistical analyses, confirm that the following items are present in the figure legend, table legend, main text, or Methods section.

n/a	Confirmed	
☐	☒	The exact sample size (*n*) for each experimental group/condition, given as a discrete number and unit of measurement
☐	☒	A statement on whether measurements were taken from distinct samples or whether the same sample was measured repeatedly
☐	☒	The statistical test(s) used AND whether they are one- or two-sided<br>*Only common tests should be described solely by name; describe more complex techniques in the Methods section.*
☐	☒	A description of all covariates tested
☐	☒	A description of any assumptions or corrections, such as tests of normality and adjustment for multiple comparisons
☐	☒	A full description of the statistical parameters including central tendency (e.g. means) or other basic estimates (e.g. regression coefficient) AND variation (e.g. standard deviation) or associated estimates of uncertainty (e.g. confidence intervals)
☐	☒	For null hypothesis testing, the test statistic (e.g. *F*, *t*, *r*) with confidence intervals, effect sizes, degrees of freedom and *P* value noted<br>*Give P values as exact values whenever suitable.*
☒	☐	For Bayesian analysis, information on the choice of priors and Markov chain Monte Carlo settings
☐	☒	For hierarchical and complex designs, identification of the appropriate level for tests and full reporting of outcomes
☐	☒	Estimates of effect sizes (e.g. Cohen's *d*, Pearson's *r*), indicating how they were calculated

Our web collection on statistics for biologists contains articles on many of the points above.

Software and code

Policy information about availability of computer code

Data collection	GDSC data was downloaded from https://www.cancerrxgene.org/. RNA-Seq by Expectation Maximization (RSEM) normalized gene expression for epithelial-based cancers was downloaded from The Cancer Genome Atlas (TCGA) database, which was accessed through the Firebrowse database using the `RTCGAToolbox' package (version 2.20.0) in R. The Total Cancer Care Dataset is collected by the H. Lee Moffitt Cancer Center and Research Institute using protocols described in Fenstermacher et al.
Data analysis	Data was analyzed using custom code which can all be found publicly available at https://github.com/jessicascarborough/cissig.

For manuscripts utilizing custom algorithms or software that are central to the research but not yet described in published literature, software must be made available to editors and reviewers. We strongly encourage code deposition in a community repository (e.g. GitHub). See the Nature Portfolio guidelines for submitting code & software for further information.

Data

Policy information about availability of data

All manuscripts must include a data availability statement. This statement should provide the following information, where applicable:

- Accession codes, unique identifiers, or web links for publicly available datasets
- A description of any restrictions on data availability
- For clinical datasets or third party data, please ensure that the statement adheres to our policy

The Genomics of Drug Sensitivity in Cancer (GDSC) dataset was accessed using R (code in the cited GitHub repository) to directly download files from the following

links, which do not require registration:
https://www.cancerrxgene.org/gdsc1000/GDSC1000_WebResources//Data/preprocessed/Cell_line_RMA_proc_basalExp.txt.zip,
ftp://ftp.sanger.ac.uk/pub/project/cancerrxgene/releases/current_release/GDSC2_fitted_dose_response_25Feb20.xlsx,
ftp://ftp.sanger.ac.uk/pub/project/cancerrxgene/releases/current_release/Cell_Lines_Details.xlsx.
The Cancer Genome Atlas (TCGA) dataset was accessed using the `RTCGAToolbox` R package (code in the cided GitHub repository) to download each disease site's RSEM normalized RNASeq V2 (labeled `RNASeq2GeneNorm`).
The data can be accessed, without registration here:
https://gdac.broadinstitute.org/.
The Total Cancer Care (TCC) dataset requires application for access, and can be found here: https://moffitt.org/research-science/total-cancer-care/.
Approval was received to use the anonymized data in this manuscript after registration, and the IRB of Moffitt Cancer Center gave ethical approval for the collection of the original data.

Human research participants

Policy information about studies involving human research participants and Sex and Gender in Research.

Reporting on sex and gender	n/a
Population characteristics	n/a
Recruitment	n/a
Ethics oversight	n/a

Note that full information on the approval of the study protocol must also be provided in the manuscript.

Field-specific reporting

Please select the one below that is the best fit for your research. If you are not sure, read the appropriate sections before making your selection.

☒ Life sciences ☐ Behavioural & social sciences ☐ Ecological, evolutionary & environmental sciences

For a reference copy of the document with all sections, see nature.com/documents/nr-reporting-summary-flat.pdf

Life sciences study design

All studies must disclose on these points even when the disclosure is negative.

Sample size	Sample sizes for gene signature extraction were based on the number of cell lines included in the GDSC dataset. In prediction modeling, there are much greater than 10 samples per covariate (a value classically used as an industry standard).
Data exclusions	Only cell lines that were not epithelial in origin were excluded from our analysis. This was decided in order to improve the translation of this pan-cancer signature, as there are many fundamental differences between carcinoma, sarcoma, and hematopoietic cancers.
Replication	Performance of CisSig was compared to 1000 null models built to ensure replication of the results. Instead of assessing the signature's performance alone in predicting IC50, this ensures that we are comparing prediction results to an appropriate null distribution of results.
Randomization	Randomization was performed throughout the analysis using the `set.seed()` function in R.
Blinding	Blinding is not relevant to this study design as groups are not allocated by to other than training/testing datasets, which were randomized using the `set.seed()` in R.

Reporting for specific materials, systems and methods

We require information from authors about some types of materials, experimental systems and methods used in many studies. Here, indicate whether each material, system or method listed is relevant to your study. If you are not sure if a list item applies to your research, read the appropriate section before selecting a response.

Materials & experimental systems

n/a	Involved in the study
☒	☐ Antibodies
☐	☒ Eukaryotic cell lines
☒	☐ Palaeontology and archaeology
☒	☐ Animals and other organisms
☒	☐ Clinical data
☒	☐ Dual use research of concern

Methods

n/a	Involved in the study
☒	☐ ChIP-seq
☒	☐ Flow cytometry
☒	☐ MRI-based neuroimaging

Eukaryotic cell lines

Policy information about cell lines and Sex and Gender in Research

Cell line source(s)	All cell line data used in our analysis is published by the Genomics of Drug Sensitivity in Cancer Database (https://www.cancerrxgene.org/)
Authentication	Per the GDSC website, "To exclude cross-contaminated or synonymous lines, a panel of 92 SNPs was profiled for each cell line (Sequenom, San Diego, CA) and a pair-wise comparison score calculated for in-house identity checking. In addition, we have confirmed the identity of our cancer cell line set against those provided by the repositories, where possible. Each of the cell lines within our core set has been tested using a panel of 16 STRs (AmpFLSTR Identifiler KIT, ABI), which includes the 9 currently used by most of the cell line repositories (ATCC, Riken, JCRB and DSMZ). STR or SNP datasets for each cell line can be accessed through the cancer cell line pages of the COSMIC database (http://cancer.sanger.ac.uk/cell_lines#)."
Mycoplasma contamination	Per GDSC website and citation, there is no mention of mycoplasma contamination testing. They state, "Cells were grown in RPMI or DMEM/F12 medium supplemented with 5% or 10% FBS and penicillin/streptomycin, and maintained at 37°C in a humidified atmosphere at 5% CO_2."
Commonly misidentified lines (See ICLAC register)	Per GDSC website, "To exclude cross-contaminated or synonymous lines, a panel of 92 SNPs was profiled for each cell line (Sequenom, San Diego, CA) and a pair-wise comparison score calculated for in-house identity checking. In addition, we have confirmed the identity of our cancer cell line set against those provided by the repositories, where possible. Each of the cell lines within our core set has been tested using a panel of 16 STRs (AmpFLSTR Identifiler KIT, ABI), which includes the 9 currently used by most of the cell line repositories (ATCC, Riken, JCRB and DSMZ). STR or SNP datasets for each cell line can be accessed through the cancer cell line pages of the COSMIC database (http://cancer.sanger.ac.uk/cell_lines#)."
